# Supplementary material for: An initial ‘snapshot’ of sensory information biases the likelihood and speed of subsequent changes of mind
Source: PLoS Comput Biol. 2022 Jan 13;18(1):e1009738. doi: 10.1371/journal.pcbi.1009738 (PMC8757993; doi:10.1371/journal.pcbi.1009738)
Supplement: S1 Text — (PDF) [file pcbi.1009738.s001.pdf]

S1 Text. Marginal effects plots and parameter estimates from the mixed-effects models.

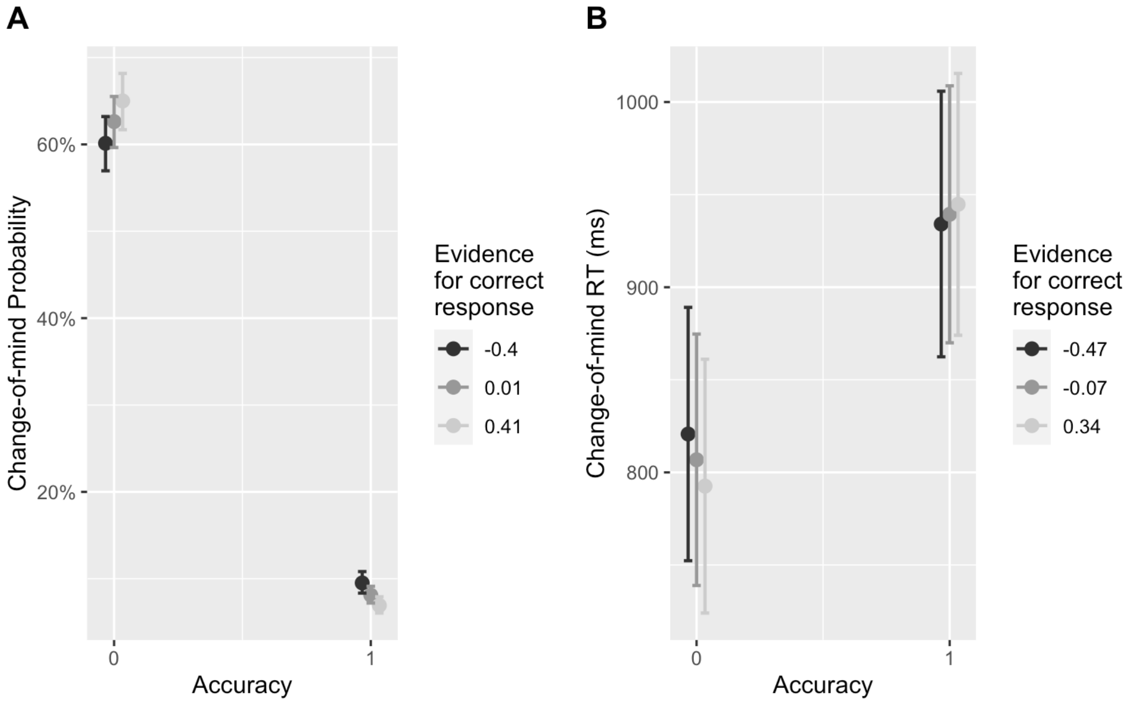

Fig A. Predicted marginal effects for the interaction between initial decision accuracy and the strength of the first frame of evidence. Panel A) shows the marginal effects from a generalised linear mixed effects model predicting whether or not a change of mind will occur. Panel B) shows the marginal effects from a linear mixed effects model predicting change of mind speed.

Table A. Mixed-effects models estimates

	Change-of-mind likelihood model			Change-of-mind RT model		
Predictors	Odds Ratios	CI	*p*	Estimates	CI	*p*
(Intercept)	1.67	1.47 – 1.90	**<.001**	804.40	736.48 – 872.31	**<.001**
Accuracy(1)	0.05	0.05 – 0.06	**<.001**	135.90	116.90 – 154.90	**<.001**
Evidence	1.29	1.13 – 1.48	**<.001**	-34.73	-56.82 – -12.64	**.002**
Accuracy*Evidence	0.50	0.41 – 0.62	**<.001**	47.85	2.54 – 93.16	**.038**
