# Supplementary material for: An initial ‘snapshot’ of sensory information biases the likelihood and speed of subsequent changes of mind
Source: PLoS Comput Biol. 2022 Jan 13;18(1):e1009738. doi: 10.1371/journal.pcbi.1009738 (PMC8757993; doi:10.1371/journal.pcbi.1009738)
Supplement: S2 Text — (PDF) [file pcbi.1009738.s002.pdf]

S2 Text. Individual-level figures and analyses.

To check that the main findings were consistent on the individual level, we plotted the reverse correlation results, separately for each participant (Fig. A), as well as the average RGB values for the brighter (Fig. B) and darker (Fig. C) stimuli respectively. In line with the results above, we found that there was generally weaker evidence favouring the initial decision on spoilt correct trials, compared to purely correct trials (for 3/4 participants; Fig. AI). For all participants we found that there was weaker evidence supporting their initial decision on corrected error trials, compared to purely incorrect trials (Fig. AJ). Similarly, for all participants there was weaker evidence supporting the initial decision for fast compared to slow corrected errors (Fig. AK). Finally, for 3/4 participants there was weaker evidence supporting the initial decision for fast compared to slow spoilt responses (Fig AL). Note that these trials were very rare (2.5% of trials for p1, 4.4% of trials for p2, 7.1% of trials for p3, and 7.5% of trials for p4), hence why there is more variability across participants.

Overall, these analyses further demonstrate that the speed and likelihood of changes of mind are associated with the degree to which the first frame of presented evidence supports participants' consequent decisions.

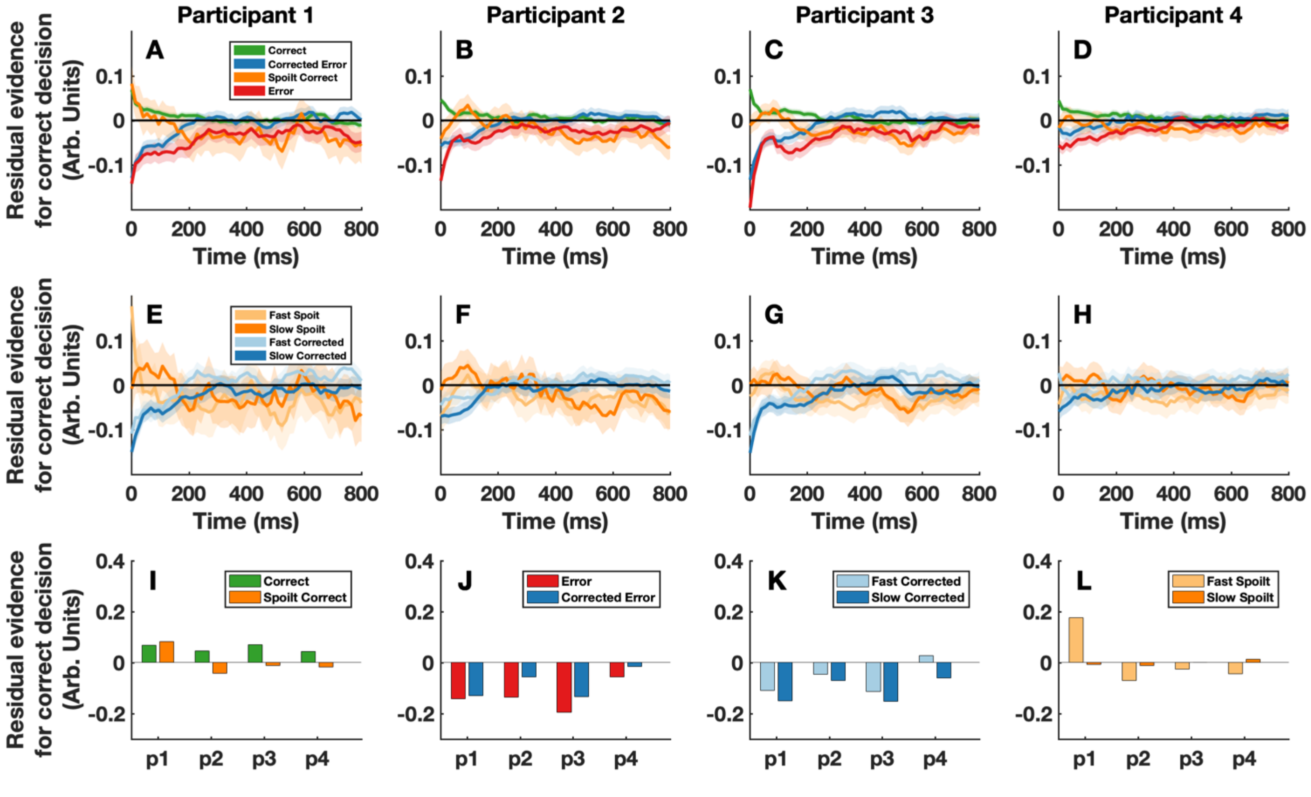

Fig A. Participant-level results. Panels A-H show the stimulus locked psychophysical kernels for each individual participant. For display purposes a moving average smoothing function with a span of 3 frames was applied. Panels I-L show the average luminance of the first frame of evidence that participants were presented with (i.e. the data for x = 0 in Panels A-H). Panel I compares correct and spoilt correct responses, panel J compares error and corrected error responses, panel K compares fast and slow corrected responses, and panel L compares fast and slow spoilt responses. In these panels, participants are represented on the x-axis.

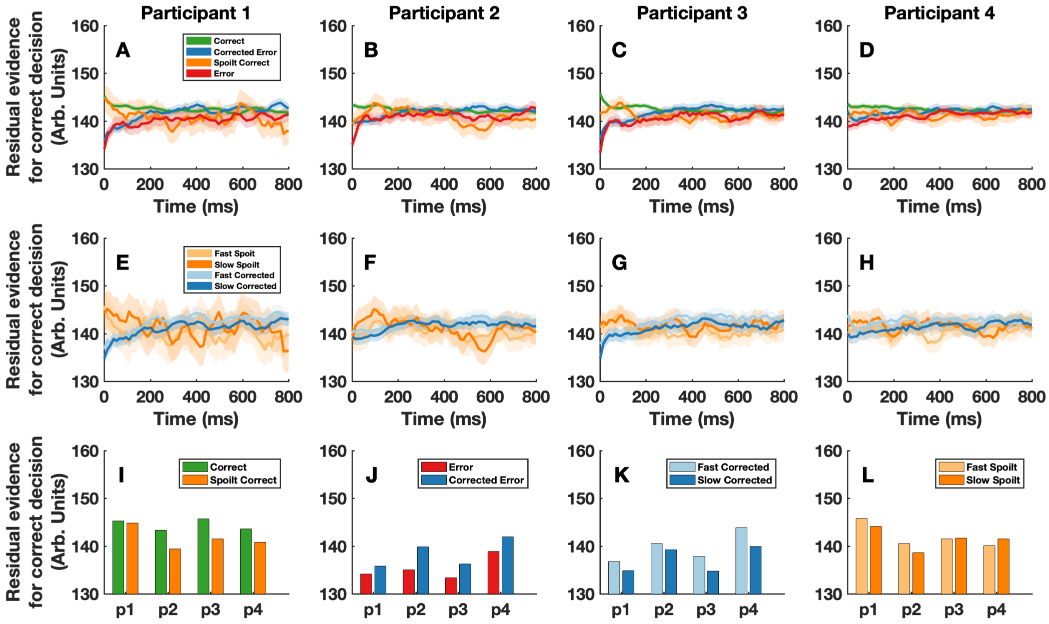

Fig B. Luminance values for the brighter (correct) stimulus. Panels A-H show stimulus locked psychophysical kernels for the brighter stimulus, for each individual participant. For display purposes a moving average smoothing function with a span of 3 frames was applied. Panels I-L show the average luminance of the brighter stimulus for the first frame of evidence that participants were presented with (i.e. the data for x = 0 in Panels A-H). Panel I compares correct and spoilt correct responses, panel J compares error and corrected error responses, panel K compares fast and slow corrected responses, and panel L compares fast and slow spoilt responses. In these panels, participants are represented on the x-axis.

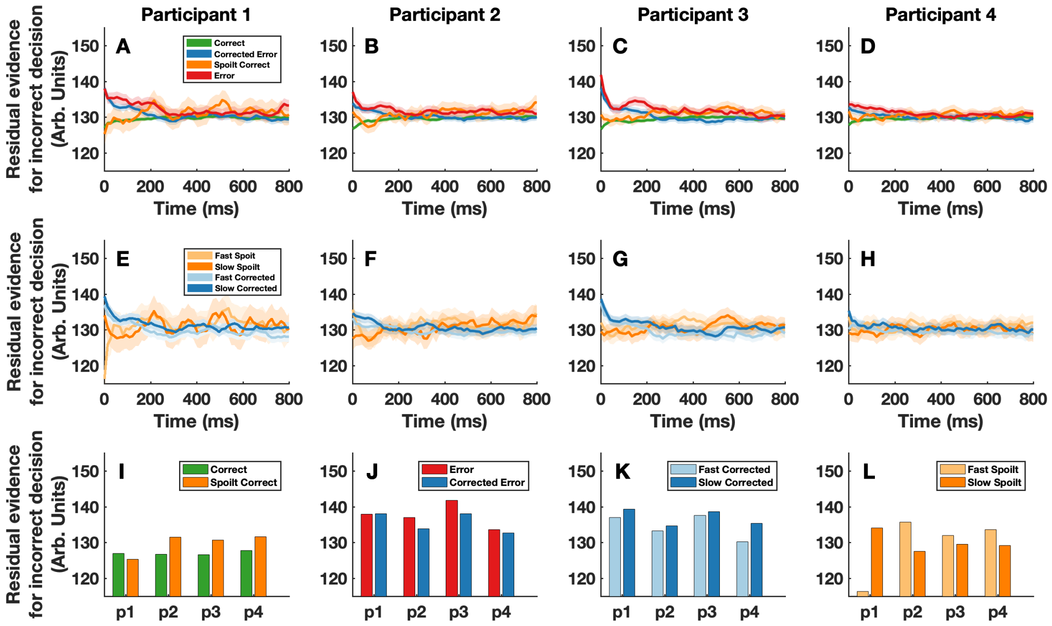

Fig C. Luminance values for the darker (incorrect) stimulus. Panels A-H show stimulus locked psychophysical kernels for the darker stimulus, for each individual participant. For display purposes a moving average smoothing function with a span of 3 frames was applied. Panels I-L show the average luminance of the darker stimulus for the first frame of evidence that participants were presented with (i.e. the data for x = 0 in Panels A-H). Panel I compares correct and spoilt correct responses, panel J compares error and corrected error responses, panel K compares fast and slow corrected responses, and panel L compares fast and slow spoilt responses. In these panels, participants are represented on the x-axis.
