# Supplementary material for: An initial ‘snapshot’ of sensory information biases the likelihood and speed of subsequent changes of mind
Source: PLoS Comput Biol. 2022 Jan 13;18(1):e1009738. doi: 10.1371/journal.pcbi.1009738 (PMC8757993; doi:10.1371/journal.pcbi.1009738)
Supplement: S3 Text — (PDF) [file pcbi.1009738.s003.pdf]

S3 Text. Model predictions and parameters.

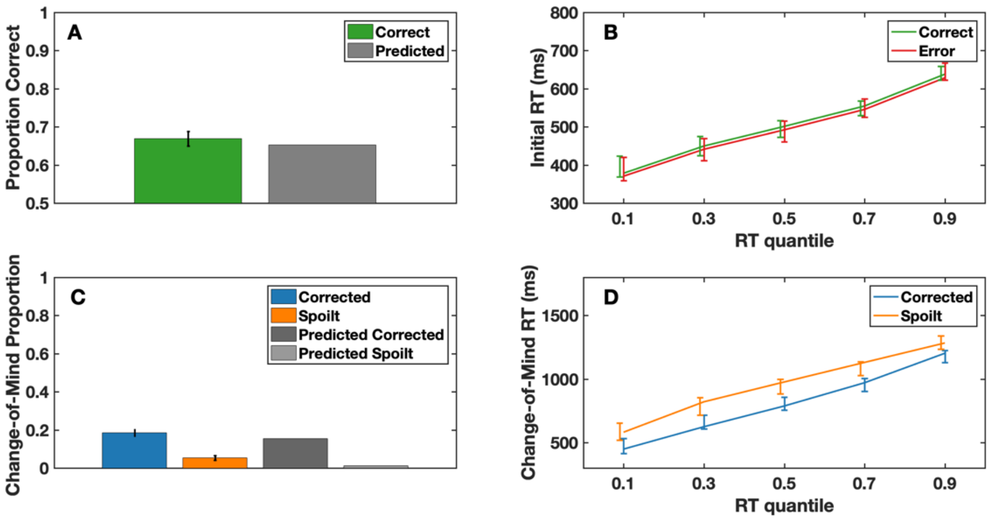

Fig A. Behavioural responses and model predictions. Panels A-D show the behavioural predictions from the model, alongside the actual data. In panels B and D, the error bars represent the 95% confidence intervals around the mean of the experimental data, whilst the lines represent the predictions from the model. In panels A and C the black error bars represent the 95% confidence interval around the mean of the experimental data, and the grey bars denote the model predictions.

Table A. Parameter estimates for the full variant of the extended diffusion decision model. *B* is the initial decision threshold, *Sz* is the range of the starting point variability, *tnd* is the non-decision time component for initial decisions, *tndVar* is the variability in the non-decision time component for initial decisions, *mu* is the mean drift rate, *theta* is the stimulus-driven within trials noise component used to model the fluctuations in the stimulus, *eta* is the endogenous across-trial drift rate variability component, *s* is the scaling parameter used to scale the internal representation of the first frame of evidence into the externally driven drift rate variability component, B_{CoM} is the distance of the change of mind threshold from the initial decision threshold, *timeOut* determines that amount of post-decisional evidence that is considered (as a proportion of the total available time), and the *slope* parameter determines the rate at which the initial evidence bias decays.

B	*Sz*	*tnd*	*tndVar*	*mu*	*theta*	*eta*	*s*	B_{CoM}	*timeOut*	*slope*
0.153	0.150	0.445	0.084	0.461	0.449	0.370	0.357	0.568	0.903	0.0001

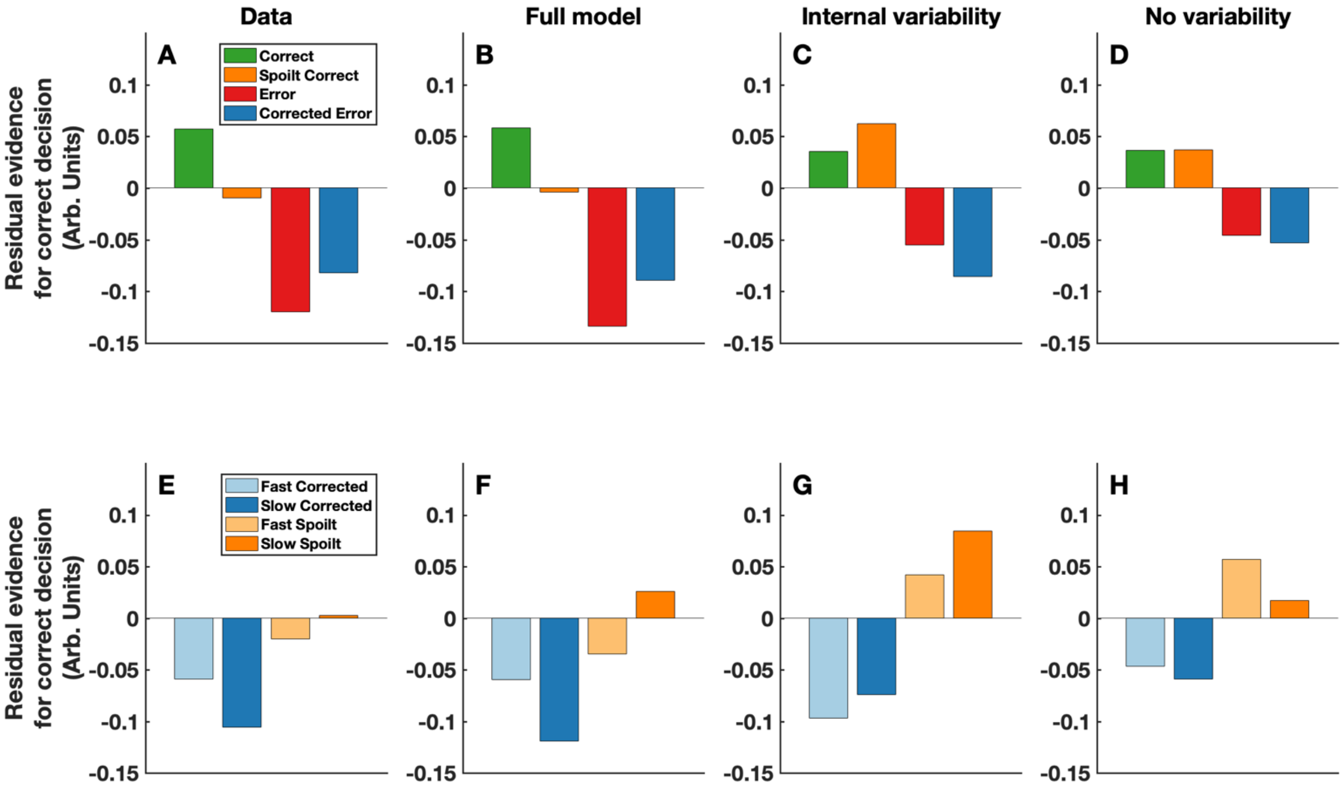

Fig B. Reverse correlation results for the first frame of evidence. This figure displays the data from Fig. 3, for x = 0 only (i.e. for the very first frame of evidence). It is clear from this figure that a combination of externally and internally determined, across-trial drift rate variability is needed to capture the weighting initial evidence across response types.
