# Supplementary material for: An initial ‘snapshot’ of sensory information biases the likelihood and speed of subsequent changes of mind
Source: PLoS Comput Biol. 2022 Jan 13;18(1):e1009738. doi: 10.1371/journal.pcbi.1009738 (PMC8757993; doi:10.1371/journal.pcbi.1009738)
Supplement: S4 Text — (PDF) [file pcbi.1009738.s004.pdf]

S4 Text. Marginal effects plot for pre-response evidence model.

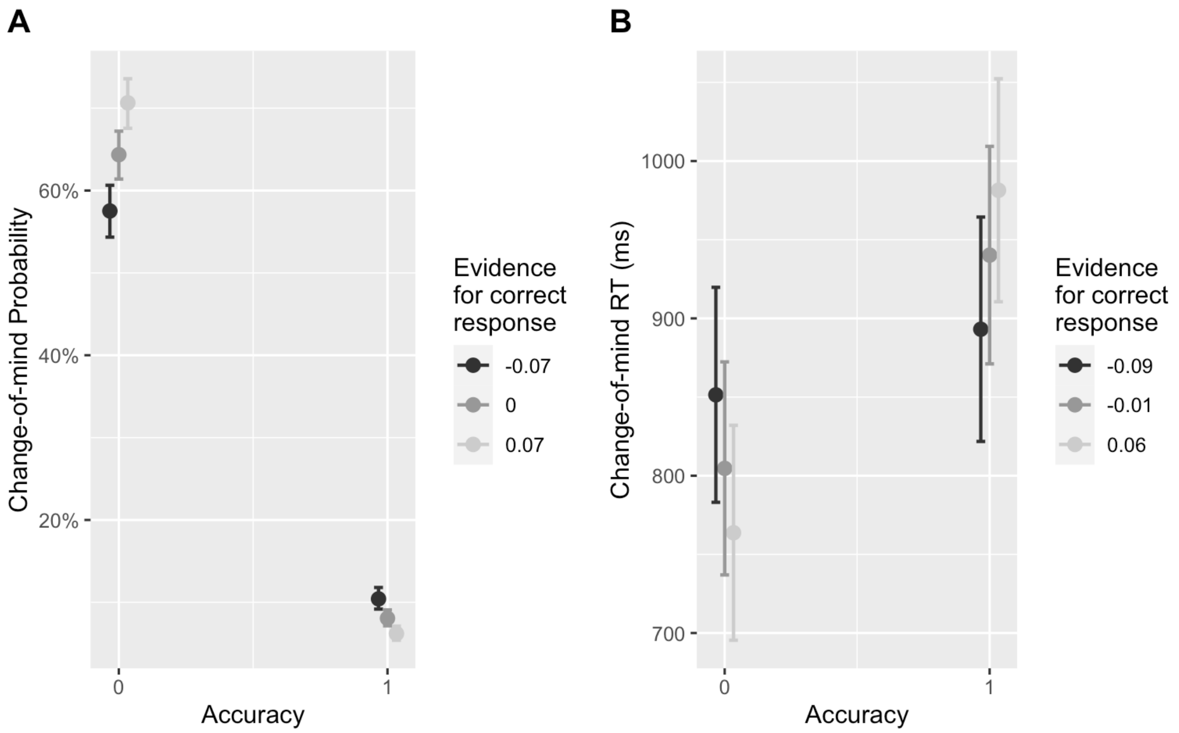

Fig A. Predicted marginal effects for the interaction between initial decision accuracy and the strength of mean pre-response evidence (excluding the initial frame). Pre-response evidence was calculated as the average of the normalized residual evidence fluctuations in the time period prior to each initial button press. Panel A) shows the marginal effects from a generalised linear mixed effects model predicting whether or not a change of mind will occur. Panel B) shows the marginal effects from a linear mixed effects model predicting change of mind speed. For both the legends, positive values indicate that the average level of pre-response evidence was in favour of the correct response. For both panels, x-axis values of 0 indicate an incorrect initial response and values of 1 indicate a correct initial response.
