# Supplementary material for: An initial ‘snapshot’ of sensory information biases the likelihood and speed of subsequent changes of mind
Source: PLoS Comput Biol. 2022 Jan 13;18(1):e1009738. doi: 10.1371/journal.pcbi.1009738 (PMC8757993; doi:10.1371/journal.pcbi.1009738)
Supplement: S5 Text — (PDF) [file pcbi.1009738.s005.pdf]

S5 Text. Auxiliary analysis of trials in which the signal favoured the incorrect response

We conducted an auxiliary analysis of the proportion of trials in which, by chance, the overall mean signal was in favour of the incorrect response, to rule out that these trials were driving our results in some way. When averaging across all timepoints, the proportion of trials in which this occurred was 0.00067 (i.e. 13 trials). However, one reason this proportion is so small is because it is calculated across the whole trial, which includes the full 1 second post-decisional time period. Given this, we ran an additional analysis on the first 600ms of each trial. We chose this time window since it approximates the maximum duration that participants would have been sampling evidence for their initial decision, given the estimated kernels in Figure 1B. Even in this time window the proportion of trials was only 2.98%. Given that the overall change of mind rate was 23.91% (18.53% corrected errors), it is unlikely that trials in which mean evidence was favouring the incorrect response were driving this behaviour. The psychophysical reverse correlation analyses further serve to demonstrate that this is unlikely. These analyses show that changes of mind were driven by stereotyped changes in information across time, where initial information supports once choice and later information supported the other. Finally, we note that random fluctuations in favour of the incorrect response are a feature of computational models we employed. This serves as further evidence that the observed effects are unlikely to be driven by trials in which mean evidence favoured the incorrect response.
