# Supplementary material for: An initial ‘snapshot’ of sensory information biases the likelihood and speed of subsequent changes of mind
Source: PLoS Comput Biol. 2022 Jan 13;18(1):e1009738. doi: 10.1371/journal.pcbi.1009738 (PMC8757993; doi:10.1371/journal.pcbi.1009738)
Supplement: S6 Text — (PDF) [file pcbi.1009738.s006.pdf]

S6 Text. Parameter estimates for 1-200 ms and 200-400 ms mean evidence models.

Table A. Mixed-effects models estimates

	Change-of-mind likelihood (Mean evidence 1-200ms)			Change-of-mind likelihood (Mean evidence 200-400ms)		
Predictors	Odds Ratios	CI	*p*	Odds Ratios	CI	*p*
(Intercept)	1.78	1.56 – 2.02	**<.001**	1.68	1.49 – 1.90	**<.001**
Accuracy(1)	0.05	0.05 – 0.06	**<.001**	0.05	0.5 – 0.06	**<.001**
Evidence	6.26	3.66 – 10.70	**<.001**	13.40	7.90 – 22.71	**<.001**
Accuracy*Evidence	0.02	0.01 – 0.05	**<.001**	0.01	0.00 – 0.01	**<.001**
Marginal R^2/Conditional R^2		0.364 / 0.367			0.370 / 0.372	
