# Supplementary material for: An initial ‘snapshot’ of sensory information biases the likelihood and speed of subsequent changes of mind
Source: PLoS Comput Biol. 2022 Jan 13;18(1):e1009738. doi: 10.1371/journal.pcbi.1009738 (PMC8757993; doi:10.1371/journal.pcbi.1009738)
Supplement: Attachment — Submitted filename: Turner_Response_to_Reviewers.docx [file pcbi.1009738.s007.docx]

**Response to Reviewers**

We thank the Editor and Reviewers for the opportunity to revise our manuscript “**An initial ‘snapshot’ of sensory information biases the likelihood and speed of subsequent changes of mind**”. We have responded to all Reviewer comments and suggestions, and are confident that we present a much improved manuscript.

In our response below, we address each of the Reviewers’ comments in turn. Reviewer comments are in bold, and our responses are in regular font. Quoted sections from the manuscript are in italics. For clarity and cross-referencing purposes, we have coded each of the Reviewers’ comments (e.g., R1C1, R2C2 etc.). Line numbers are used to indicate the location of specific changes in the revised manuscript. Figures which are included in the response letter but not in the revised manuscript are prefixed with the letter R. Figures included in quoted sections of the revised manuscript retain their manuscript-specific figure number. All changes in the revised manuscript are highlighted in yellow.

**Reviewer #1**

**In this paper, the authors explore how the quantity of evidence accumulated before making a choice continues to impact the likelihood of changing one’s mind later on. Participants were presented two stimuli fluctuating in luminosity around two sets mean values. On each trial, they had to decide which of the two stimuli was the brightest. After the initial choice, the stimuli continued to be displayed, leaving the possibility to participants to change their mind. Reverse correlating the stimuli fluctuations to the choice revealed that early evidence had a long lasting impact on choice, as well as on the likelihood and speed of changes of minds. The authors used an extension of the Drift Diffusion Model to explain how early evidence can be used as a snapshot to which further evidence is compared and used to decide to change one’s mind.**

**The study is well conducted and certainly provides interesting new evidence on reversals of decisions. The paper is well written and easy to read, despite the complexity of the modelling and the statistics used. The main effect reported in the paper, namely the extension of primacy bias beyond choice to the timing and likelihood of changes of minds, is novel and certainly would constitute a significant contribution to the field. I also found the modelling approach appropriate and interesting, allowing to capture the behavioural data in a comprehensive manner.**

We thank the Reviewer for their detailed summary and positive appraisal of our work.

**R1C1. My main concern however is the generalizability of the main finding of a long lasting effect of an early snapshot of evidence on changes of mind. Although primacy effects have been reported in the literature, such effect has not been observed on previous studies investigating changes of minds. Considering the novelty of the effect, it seems necessary to test further its robustness and explore alternative hypothesis. In particular, although reverse correlation is a powerful tool to understand the dynamics of decisions process, it is also notoriously hard to interpret and can lead to biases in interpretation. As the whole paper is centred on reverse correlation to prove the presence of a primacy effect on changes of mind, I find that additional analysis and/or experiments would be necessary to justify its existence. I list below additional comments and concerns.**

We appreciate the Reviewer’s concerns around the generalisability of the primacy effect, and agree that they are important to consider. However, for reasons which we will briefly summarise in the following paragraph, and will explain in greater detail below, while it may only be possible to observe the primacy effect in certain experimental paradigms, we believe that the existence of this effect nevertheless has important general theoretical implications for our understanding of the cognitive processes underlying perceptual decisions and changes of mind.

The dominant view within cognitive neuroscience/psychology is that relatively general computational processes underlie simple perceptual decisions across a range of different tasks and modalities (e.g., luminance judgements, motion judgements, tactile judgements etc.). While the nature of the information feeding into this process may differ across domains, models of this process, including those which account for changes of mind (e.g., Resulaj et al., 2009), are taken as general rather than domain specific. This view is not unfounded, as common neural signatures of core decision processes, such as evidence accumulation, can be observed across different modalities/tasks – suggesting a relatively general decision mechanism (e.g., O’Connell et al., 2012; O’Connell & Kelly, 2015). In our response to R1C3 below, we will argue that the primacy effect we are reporting may only be observable in specific paradigms (e.g., in luminance judgement tasks but not in motion judgement tasks). Nevertheless, given the view that a general process with task-specific inputs underlies perceptual decisions and changes of mind, the fact that such a primacy effect can even be observed has important implications for our understanding of the general process underlying perceptual changes of mind. In particular, it tells us that the pre- and post-decisional dynamics of this process are not distinct, and do interact–counter to dominant perspectives (Resulaj et al., 2009).

In our response to the Reviewers comments below, we will expand upon these points in more detail.

**R1C2. Test of the primacy effect: As I explained above, I believe further tests should be performed to understand the relative effect of the initial snapshot of evidence compared to future evidence on changes of mind. In particular, I think different GLMs including alternative predictors such as the overall pre-decisional signal strength or the evidence immediately preceding the response should be tested to establish the best model predicting the occurrence of changes of minds.**

We have now run additional analyses which are reported in the Supporting Information of the revised manuscript. In these analyses we calculated the average pre-response residual evidence (excluding the initial frame) for each trial. We then fit a generalized linear mixed effects model to predict the probability of a change of mind occurring from the pre-response evidence. These analyses show that pre-response evidence, besides the initial frame, strongly predicts the speed and likelihood of changes of mind. This demonstrates that the initial frame of evidence is neither the sole, nor necessarily the best, predictor of perceptual changes of mind.

Below we first report these new analyses as they are reported in the revised manuscript. Then, we explain why these analyses, whilst helping to contextualise the magnitude of the primacy effect, do not challenge our overall conclusions.

The additional analyses are now reported in S4 Appendix of the Supporting Information:

*‘To examine the overall effect of pre-response evidence on change-of-mind likelihood, we fit a generalized linear mixed effects model to predict the probability of a change of mind occurring from the average pre-response evidence. For our measure of pre-response residual evidence, we excluded the initial frame as this is analysed in the main results. This also allowed us to derive an estimate of the relative magnitude of the influence that the initial frame of evidence has on change-of-mind likelihood, as compared to all other pre-response evidence. This analysis revealed a significant interaction between initial decision accuracy and pre-response evidence strength on the probability of a change of mind occurring (likelihood ratio test, χ² (1) = 192.36, p < 2.20 x 10^-16^; see Fig. S7A). This indicates that when pre-response evidence strongly supported participants’ initial decisions, then changes of mind were less likely to occur.*

*To formally test the effect of pre-response evidence on change of mind latency, we fit a linear mixed effects model to predict the speed with which changes of mind occurred. This revealed a significant interaction between initial decision accuracy and pre-response evidence strength on change-of-mind speed (likelihood ratio test, χ²(1) = 82.36, p = 2.20 x 10^-16^; see Fig. S7B).*

***Fig S7.*** *Predicted marginal effects for the interaction between initial decision accuracy and the strength of mean pre-response evidence (excluding the initial frame). Pre-response evidence was calculated as the average of the normalized residual evidence fluctuations in the time period prior to each initial button press. Panel A) shows the marginal effects from a generalised linear mixed effects model predicting whether or not a change of mind will occur. Panel B) shows the marginal effects from a linear mixed effects model predicting change of mind speed. For both the legends, positive values indicate that the average level of pre-response evidence was in favour of the correct response. For both panels, x-axis values of 0 indicate an incorrect initial response and values of 1 indicate a correct initial response.’*

These analyses demonstrate that the initial frame of evidence is not the sole predictor of changes of mind. Indeed, examining the participants’ psychophysical kernels in Fig. 1 of the main manuscript, it is clear that changes of mind are driven by stereotyped changes in sensory information across many frames, rather than just the initial frame of evidence.

To clarify our opinion on the relative importance of the primacy effect, we did not intend to give the impression that the initial evidence is the *best* predictor of future changes of mind. Indeed, we believe that overall pre-response evidence and post-response evidence are very important in determining whether a change of mind will occur. As we state in the Results section (Lines 144-147):

*‘… However associations between stimulus evidence strength and decision behaviour were by no means restricted to just the first frame. For illustrative purposes, we therefore also plot the results of frame-by-frame t-tests to compare differences in residual evidence throughout the trial (see Fig. 1).’*

We now make this point even more explicitly in S4 Appendix of the revised manuscript:

‘*Importantly, these analyses show that the initial frame of evidence is neither the sole, nor the best, predictor of change of mind behaviour. Indeed, consistent with the general patterns revealed by the psychophysical reverse correlation analysis, these analyses highlight how changes-of-mind tend to arise due to stereotyped changes in the sensory evidence across time, whereby initial evidence supports the participants first decision, and later evidence supports the alternative choice, driving the change of mind.’*

Critically however, as we have argued above, the fact that the earliest frame of evidence *is even able to influence change of mind decisions* has important theoretical implications, regardless of the magnitude of this effect. In particular, it tells us that the pre- and post-decisional dynamics of the decision process are not distinct. Given that this runs counter to the most influential model of perceptual changes of mind (Resulaj et al., 2009), we think this is an important effect which will be of general interest.

**R1C3. Generalizability of the primacy effect to different paradigms: One important question raised by these findings is how likely such primacy effect is going to be observed for other types of decisions and stimuli. The authors do not discuss whether such effect is specific to the luminance task they used. Indeed, luminance could be seen as a stimulus feature that by definition requires less temporal integration than motion perception for instance, explaining why early momentary evidence might have such strong influence on choice and changes of mind. I would suggest to test whether such pattern of results would be observed in RDK stimuli or other stimulus discrimination task. This is important to understand the scope of the findings. A paragraph on this should also be added in the discussion.**

We agree that it is important to consider whether our findings will generalise to different paradigms. Below we will explain why it is possible that the primacy effect we are reporting may only be observable in certain low-level tasks which involve stimuli that require minimal temporal integration. However, we will then reiterate why the very existence of this effect nevertheless tells us something general about the structure of the computational process underlying perceptual changes of mind.

As the Reviewer is no doubt aware, the majority of previous studies investigating changes of mind have used random dot kinematograms (RDKs) as the stimulus. This design choice is important as it limits one’s ability to examine the processing of very early stimulus information due to the 50-150 ms lag induced by motion energy filtering, and the initial filter build-up time. Below we present pilot data from our lab which highlights this issue.

It was in light of this issue that we initially decided to use a luminance judgement task, with independent information at each frame. Similarly, here we have decided to not run the full suggested study, but instead address this question in detail within the Discussion section of the revised manuscript.

To better illustrate the issue with RDK stimuli, below we include a plot of pilot data from our lab in which participants (n = 8) performed a similar change of mind experiment with RDKs (Figure R1). In this experiment, participants had to indicate whether the overall direction of dot motion was to the left or the right. Participants made their responses by squeezing on handheld dynamometers (force sensitive handles) until a force threshold was reached. Changes of mind could occur if participants initially began squeezing on one handle, but before reaching the force threshold, switched to squeezing the alternative handle.

**Figure R1. Trial diagram and pilot data from an experiment involving RDK stimuli. A)** Participants viewed a fixation cross and were then presented with an RDK. They had to judge whether the dots were drifting leftwards or rightwards. Participants made their responses by squeezing on handheld dynamometers which would ‘fill’ the response bar either leftwards or rightwards. When a specific force threshold was reached the response would be recorded. Participants were able to change their mind before this point, by squeezing the other dynamometer. **B)** Motion energy filtering was used to extract a moment-to-moment estimate of the amount of residual evidence in favour of the correct, relative to the incorrect, decision (this was done using the motion energy toolbox from Urai & Wimmer, 2016 and filter parameters from Kiani et al., 2008). In this figure, positive values indicate relatively more evidence in favour of correct decision. Psychophysical kernels for four trial types are plotted. The average motion energy for trials in which participants made a correct decision and did not change their mind are plotted in blue. The average motion energy for trials in which participants made an incorrect decision and did not change their mind are plotted in purple. Finally, the average motion energy for trials in which participants made an incorrect decision and then changed their mind (‘corrected error’ trials), either quickly (faster than the mean change-of-mind time) or slowly (slower than the mean change-of-mind time) are plotted in red and green, respectively. For this pilot data there were too few ‘spoilt correct’ trials to construct reliable psychophysical kernels. The shaded areas indicate 1 SEM.

The patterns in Figure R1 are generally consistent with our observations in the luminance judgement task. In particular, correct trials tend to be associated with more evidence in favour of the correct response, error trials are associated with less evidence in favour of the correct response, and change of mind are associated with a shift in evidence from one response to another. The speed of this shift is also associated with the change of mind response time. Critically however, by examining the early time windows of Figure R1, we can see how it is effectively impossible to examine the weighting of very early stimulus information with RDK stimuli, as the motion energy filters require at least ~50 ms to build up sufficiently. Moreover, given the temporal properties of these filters, even if differences in the psychophysical kernels are observable in early time windows, one cannot rule out the possibility that these are driven by differences at later timepoints which are smeared forwards in time. These issues are likely why past studies employing RDK stimuli have not reported the primacy effect we observed.

Charles and Yeung (2019) examined the weighting of information underlying change of mind decisions in the context of a numerosity judgement task, which does not suffer from the same filtering issues. We agree that it would be interesting and worthwhile to examine whether the initial frames of information in the context of numerosity judgement tasks also show evidence of a primacy effect, as this would help to test the generalizability of our current findings. However, to our knowledge the data from this study are not publicly available, prohibiting such a re-analysis.

More generally, as the reviewer notes, it is possible that the effect we report may only be observable in the context of low-level visual tasks which do not require much temporal integration. However, as we have argued above, given the view that a general computational process, with task-specific inputs, underlies perceptual decisions and changes of mind in simple perceptual tasks (Resulaj et al., 2009), then the fact that such an effect can even be observed is of theoretical importance.

We fully agree with the reviewer that this is an important question that should be addressed in future research. An exploration of all possible paradigms in which change of mind can occur however, is beyond the scope of our study. We now discuss these issues in greater detail in the Discussion section of the revised manuscript, and mention the possibility that effects might be constrained by features of specific paradigms (Lines 312-325):

*‘… Other studies investigating perceptual changes of mind have typically employed random dot motion tasks, making it impossible to examine the frame-by-frame processing of early sensory information in these experiments due the 50-150 ms lag induced by motion energy filtering. By employing a luminance discrimination task, we were able to circumvent this issue and present clear evidence that change-of-mind behaviour is affected by even the very earliest (pre-decisional) information one receives. However, it may be the case that the ‘primacy effect’ we observed is only detectable in the context of low-level visual tasks which require minimal temporal integration. If future work identified a differential use of information for change of mind, depending on the features of evidence integration, this would in itself advance our understanding of decision dynamics. However, given the dominant view that relatively general computational processes underlie simple perceptual decisions and changes of mind across different perceptual tasks (O’Connell et al., 2012; O’Connell & Kelly, 2015; Resulaj et al., 2009), the effect we demonstrate also has general implications. In particular, it demonstrates that the pre- and post-decisional dynamics are not distinct, and do in fact interact, counter to dominant perspectives (e.g., Resulaj et al., 2009).’*

**R1C4. Another concern is the fact that the authors used constant means for the low and high luminance stimuli. Therefore, it is possible for the participants to perform the task not as a discrimination task but as a detection task, learning to detect the presence or absence of the high luminance stimulus on one side of the screen only. Indeed, the presence of the primacy effect could be explained by an early “detection strategy” where the presence of high luminance signal at the onset of the trial in one of the stimuli would lead participants to believe they have detected the presence of the high luminance stimulus, while later accumulation of evidence would switch to a comparison of the evidence of the 2 stimuli. To avoid participants using such strategy, it would have been interesting to vary in the design the overall mean luminance of the two stimuli, while keeping the difference in luminance between them constant. That would have allowed to determine whether the primacy effect on changes of mind is only a by-product of the task design and participants optimizing their choice process for that design. I would suggest such experiment is added to the design and this hypothesis is discussed in the paper too.**

We agree that it is important to consider whether participants might have adopted a ‘detection’ rather than ‘discrimination’ strategy. Below, we will first outline why we chose to use constant means for the stimuli rather than varying their overall brightness. Then, we will explain why, despite this decision, we believe it is unlikely that participants adopted an ‘early detection’ strategy.

The reason we chose to use constant means for the two stimuli was to ensure maximum practicable statistical power for the psychophysical reverse correlation analyses. Because the dynamics of the decision process will change if the stimulus values are changed (even if just their absolute values are manipulated while their difference is held constant, e.g., Turner et al., 2020; Ratcliff et al., 2018) it is inadvisable to construct psychophysical kernels across different stimulus conditions. Indeed, many previous studies which have employed this analysis technique ended up analysing data from just a single stimulus condition, even if data from multiple conditions were available (e.g., Okazawa et al., 2018). As such, and with the goal of estimating participants’ psychophysical kernels as accurately as possible, we chose to include just one stimulus condition as each additional condition would have effectively halved the number of useable trials.

The reason why we believe it is unlikely that participants adopted an ‘early detection’ strategy in our experiment is because of the magnitude of the stimulus variability relative to their mean difference. Below we have plotted the two distributions from which the frame-by-frame luminance values were drawn to illustrate this.

**Figure R2.** The distributions from which the moment-to-moment luminance values for each stimulus were drawn. Both are truncated (1 SD) normal distributions with a standard deviation of 55.

Critically, both the stimulus distributions were truncated with the express purpose of limiting the possibility of extreme luminance values, which may have prompted the use of an ‘early detection’ strategy (this decision was made following Ratcliff et al., 2018). Given the high degree of overlap between the two distributions, we argue that it would not have been adaptive for participants to make split second judgements on the basis of the initial frame. Nevertheless, as we cannot, with absolute certainty, rule out this possibility we have included a paragraph on this issue in the Discussion section of the revised manuscript (Lines 366-382):

*‘We chose to keep the means of the two stimuli fixed, and did not systematically vary their overall luminance. This was to maximise the number of useable trials in the psychophysical reverse correlation analyses. Because the dynamics of the decision process change if the stimulus values are changed (even if just their absolute values are manipulated while their difference is held constant, e.g., Turner et al., 2020; Ratcliff et al., 2018), each additional stimulus condition would have effectively halved the number of useable trials. However, one concern which stems from this decision is that participants may have adopted a ‘detection’ rather than ‘discrimination’ strategy. That is, participants may have initially focused on just one stimulus and made an initial ‘detection’ decision as to whether this was the brighter/darker stimulus. Then subsequently, they may have evaluated this via a more comparative judgement process. However, two factors decrease the likelihood of this possibility: firstly, the high degree of variability within the stimuli, relative to their mean difference, and secondly, the fact that both stimulus distributions were truncated at 1 SD from the mean to avoid extremely bright or dark stimulus values. Moreover, since participants’ initial response times were well fit by a model which assumed a bounded evidence accumulation process, a hybrid-decision process does not offer the most parsimonious account of our findings. Nevertheless, future research is ultimately needed to fully examine the possibility of participants adopting a hybrid decision process in certain tasks or contexts.’*

**R1C5. One important potential limit of reverse correlation analysis is to neglect the effect of signal mean on choice, as it focusses only on fluctuations above and below the stimulus mean. However, the mean difference between the two signals has certainly an effect on choice. I would suggest that the authors provide supplementary figures showing the unaltered values of signal fluctuations in each of the luminance stimuli in a supplementary figure. That would allow to understand better what is the actual signal feeding into the decision and changes of mind. In particular, this would allow to determine whether the two stimuli have a symmetrical effect on choice and changes of minds.**

We agree that it is interesting and worthwhile to consider the unaltered values of the two stimuli seperately. In S2 Appendix of the revised manuscript we now include Figures S3 and S4 which display the average moment-to-moment stimulus values for the brigther and darker stimuli, respectively.

**Figure S3.** Luminance values for the brighter (correct) stimulus. Panels A-H show stimulus locked psychophysical kernels for the brighter stimulus, for each individual participant. For display purposes a moving average smoothing function with a span of 3 frames was applied. Panels I-L show the average luminance of the brighter stimulus for the first frame of evidence that participants were presented with (i.e. the data for x = 0 in Panels A-H). Panel I compares correct and spoilt correct responses, panel J compares error and corrected error responses, panel K compares fast and slow corrected responses, and panel L compares fast and slow spoilt responses. In these panels, participants are represented on the x-axis.

**Figure S4.** Luminance values for the darker (incorrect) stimulus. Panels A-H show stimulus locked psychophysical kernels for the darker stimulus, for each individual participant. For display purposes a moving average smoothing function with a span of 3 frames was applied. Panels I-L show the average luminance of the darker stimulus for the first frame of evidence that participants were presented with (i.e. the data for x = 0 in Panels A-H). Panel I compares correct and spoilt correct responses, panel J compares error and corrected error responses, panel K compares fast and slow corrected responses, and panel L compares fast and slow spoilt responses. In these panels, participants are represented on the x-axis.

From these figures, we can see that the effects are generally quite symmetric, with both the luminance of the brighter and darker square influencing participants’ initial decisions and changes of mind. For the rarest trial type, spoilt-correct trials, there is admittedly a bit less consistency across participants; however, this is not particularly surprising given the limited numbers of trials. Overall however, the general pattern of differences across the stimuli and trial types clearly shows that the two stimuli have symmetrical effects on participants’ decisions within this task.

In the results section of the revised manuscript, we now signpost these figures as follows (Lines 178-179):

*‘These effects were consistent on the individual level and were symmetric across the two stimuli (reported in S2 Appendix).’*

**R1C6. Using the same line of reasoning, I would suggest that the authors provide further analysis to determine the proportion of trials where, by chance, the overall signal favoured the incorrect response. Is it possible that they drive the reverse correlation effect and some very types of changes of mind? These trials may be difficult to analyse and classify as correct or incorrect, and should probably be removed from further analysis or analysed separately.**

The proportion of trials in which, by chance, the overall signal was in favour of the incorrect response was only 0.00067 (i.e. 13 trials). However, one reason this proportion is so small is because it is calculated across the whole trial, which includes the full 1 second post-decisional time period. Given this, we ran an additional analysis on just the first 600ms of each trial. We chose this time window since it approximates the maximum duration that participants would have been sampling evidence for their initial decision, given the estimated kernels in Figure 1B of the original manuscript. Even in this time window the proportion of trials was still only 2.98%. Given that the overall change of mind rate was 23.91% (18.53% corrected errors), it seems unlikely that trials in which evidence is by chance favouring the incorrect response are driving this behaviour. Indeed, the psychophysical reverse correlation analyses further serve to demonstrate that this is not the case. These analyses show that changes of mind are driven by stereotyped changes in information across time, where initial information supports once choice and later information supports the other.

It is worth noting that many, if not all, previous studies that investigated changes of mind almost certainly also had a small percentage of trials in which overall evidence supported the incorrect choice, since the use of time-varying, noisy stimuli is not unique to our study. In fact, certain studies even restricted their psychophysical reverse correlation analyses to just those trials in which there was zero mean evidence (e.g., Okazawa et al., 2018). In this case, any negative going fluctuations would have been in favour of the ‘incorrect’ response. As such, the existence of trials in which the net evidence supports the incorrect response does not invalidate or challenge the conclusions that can be drawn from psychophysical reverse correlation analyses. Moreover, given the fact that random fluctuations that support the incorrect response are a feature of computational model we used, we do not see how such trials are difficult to analyse/interpret. As such we do not believe it is necessary to remove them or analyse them separately.

**R1C7. Another question that arises from the present finding and modelling developed is what exactly the primacy effect reported means in terms of weight given to each sample of information. In particular, while the reverse correlation can be interpreted as early evidence having stronger effect on changes of mind and therefore being weighted more in the evidence accumulation process, the converse interpretation is also plausible. Namely, the signal fluctuations in the early presentation of the stimulus are weighted less and therefore need to be stronger to have an impact on changes of mind, which is why they appears as deviating from the mean in the reverse correlation time-series. Can the authors comment/discuss this?**

We agree that it is important to consider alternative explanations for the effects we observed in the psychophysical kernels, such as a down-weighting of initial evidence. To address this specific point, we simulated a version of the DDM in which the drift rate was constant across trials, but the initial frame of evidence was down-weighted. We have plotted the resulting simulated kernels (Fig. R3).

**Figure R3. Simulated kernels from a model in which initial evidence is down-weighted.** Panels A & C show psychophysical kernels constructed from the group level experimental data. Panels B & D show psychophysical kernels constructed for a model in which drift rate did not vary across trials and where the initial frame of evidence was down-weighted, i.e. scaled by a factor of 0.5, relative to the other evidence within the trial.

From Figure R3, we can see that a down-weighting of initial evidence causes the initial sections of the kernels to shift towards the mean (i.e. bend inwards towards zero). Broadly speaking, this finding is consistent with the work of Okazawa et al. (2018) who examined the impact of various changes in the dynamics of accumulator models on the outcome of psychophysical reverse correlation analyses. For our purposes, the most relevant results from this paper are those which pertain to modulations in the relative balance of leak and inhibition within the decision process. When there is more leak than inhibition, early fluctuations will effectively be down-weighted and thus tend to have less of an influence. Considering Figure 6K of Okazawa et al. (2018), when early evidence was down-weighted (via a dominance of leak over inhibition), they also observed the kernels deviating in the opposite direction to the primacy effect we report. Given this, and the simulation results we presented above, we believe it is unlikely that the kernel effects we observed can be explained by a down-weighting of initial evidence alone.

As a final note, we acknowledge (and agree with) the general point that Reviewer 1 is making here about the fact that the interpretation of psychophysical reverse correlation analyses is notoriously difficult, and should be approached with caution. It was this fact which prompted us to adopt the gold-standard approach of interpreting psychophysical reverse correlation effects via model-based simulations (see Okazawa et al., 2019). Ultimately however, it is possible that some complex alternative mixture of effects within an accumulation model may offer an alternative explanation for the kernel effects we observed, and so we are not claiming to have discovered the ‘one true model’ for the current data – such a claim is beyond the scope any modelling paper. Instead, we are simply aiming to provide a coherent account of the novel effects we have observed, in the hope of fostering further exploration in this area.

To better outline our views on this, we have expanded our original discussion of this general point in the revised manuscript (Lines 349-365):

‘*One limitation of the current modelling approach is that, while our variant of the extended DDM was able to predict the weighting of the earliest frames of evidence (i.e. the experimental effect of interest), it did not perfectly predict all observed patterns in the psychophysical kernels. For example, it predicted sharper peaks in the kernels around the time at which changes of mind occurred (compare Fig. 1 C & F to Fig. 2 C & F). This discrepancy may simply be due to the fact that the model was not directly fit to the psychophysical kernels. Alternatively, this may be a general limitation of the modelling framework or specific simplifying assumptions we adopted. For example, we assumed that the distributions of non-decision times for initial and change of mind responses were identical and were normally distributed. However, recent work has questioned whether alternative distributions of non-decision times might better explain behavioural data [18]. Ultimately, our modelling approach is constrained by the knowledge and data that are currently available to us and it is possible that future work may identify some complex alternative mixture of effects/assumptions within an evidence accumulation framework which offers a different explanation for the psychophysical kernel we observed. As such, we are not claiming to have necessarily discovered the ‘one true model’ for the current data, but instead, are simply aiming to provide a coherent account of the novel effects we have observed, with the goal of fostering further exploration in this area.*’

**R1C8. Reverse correlation methods: It is unclear from the methods how the analysis dealt with the absence of data points in the reverse correlation time-series. Presumable in the stimulus locked graph later time-points are less populated? Similarly, in response-locked time-series, early time points are less populated. The authors should report how they dealt with the change in number of trials forming the reverse correlation time-courses and how it could have affected their results.**

The reviewer is correct that certain time points will have fewer trials, due to variability in response times. We in fact already dealt with this issue in our analyses, but appreciate the opportunity to clarify this and make it explicit. We have added the following sentence to the Method section (Lines 468-470):

*‘For the response-locked and change-of-mind-locked kernels, time points where there were fewer than 100 trials were excluded, to avoid noisy estimates.’*

Note that we applied this threshold for only the response-locked and change-of-mind-locked kernels. For the stimulus-locked kernels there were no time points in which data was missing, given that the post-decisional processing period was 1 second and the plotted time-period was 0-800ms.

**R1C9. In Figure 3D&H, the authors report no differences in the psychophysical kernels for the first frame of evidence in the main text. However, a clear effect is observed on the figure. Can the authors explain?**

We appreciate the Reviewer raising this question, and in hindsight can see how this is a confusing detail which needs clarification. We have added the following text to the caption of Figure 3 to explain these apparent differences (Lines 282-291).

*‘We note the slight apparent differences between the kernels in the first frame of Fig 3D & H, the largest being between fast and slow spoilt correct responses. These are not indicative of a true effect and arise due to the relative rarity of these trial (e.g., the rarity of spoilt correct responses). Across repeated simulations of the model, these differences are not consistent and will average to zero. Importantly, these differences highlight two points which it is important to be mindful of when conducting and interpreting psychophysical reverse correlation analyses. Firstly, they demonstrate the importance of aiming for maximum practicable statistical power, given the inherent noisiness of this technique. Secondly, they highlight the importance of not placing strong weight on single effects, but rather interpreting collective patterns of differences which are consistent across individuals.’*

**Reviewer #2**

**In this experiment, the authors examine changes of mind in a simple perceptual decision. They find that the early evidence (even the very first frame of evidence) has a subsequent influence on both the likelihood of a subsequent change-of-mind, and the speed at which that change-of-mind occurs. The authors propose a variant on the extended diffusion decision model in which initial sensory information has a slowly decaying bias on subsequent evidence accumulation.**

**This is a very interesting paper on an important topic. The novel approach to investigating the influence of initial information and data from this approach are a valuable contribution to the field. This also provides some important updates to an influential model of perceptual decision-making and changes-of-mind.**

We thank the Reviewer for their positive appraisal of our work.

**R2C1. One key difference that might warrant at least a bit of discussion - the current approach uses a discrete binary response (a keypress) whereas a number of the studies cited in the introduction use a continuous response such as cursor movement, joystick movement, hand movement, etc.  In those studies, changes-of-mind were often defined as a change in movement path. This may not be a major issue in interpreting the present data but it may be worth addressing somewhere in the manuscript - it is possible that decision-making processes would differ when continuous movements (which more easily allow for the initiation of movement before an initial decision is really finalized; e.g., Gallivan & Chapman, 2014) are involved.**

We agree that this is an important point to consider. As such, we now include the following paragraph in the Discussion section of the revised manuscript (Lines 383-398):

*‘We also chose to record responses in a binary rather than continuous fashion. This allowed us to precisely measure the onset times of change-of-mind responses, adding further constraint on the computational models. However, continuous response measures, such as movement trajectories, have been employed in a number of past studies (Burk et al., 2014; Moher & Song, 2014; Resulaj et al., 2009; van den Berg et al., 2016) and have the advantage of allowing for changes of mind to be observed in a potentially more graded fashion. One question which arises in light of this, is whether this may have led to slightly different decision-making behaviour. For example, when continuous response measures are employed, participants may adopt different strategies because movements can be initiated before a strict decision has been finalized (Gallivan & Chapman, 2014). However, the general pattern of changes of mind we observed in this study, and in another recent study, which also employed a binary response measure (Turner et al., 2020), mirrors those observed in studies employing continuous response measures (Burk et al., 2014; Moher & Song, 2014; Resulaj et al., 2009; van den Berg et al., 2016). In particular, across all these studies changes of mind were more common following errors and were driven by stereotyped shifts in stimulus evidence. These commonalities suggest a common underlying process, in line with the dominant view that a general process explains changes of mind across perceptual decision tasks (Resulaj et al., 2009). Ultimately however, further research is needed to properly uncover the degree to which decision-making processes differ across binary and continuous responses.’*

**R2C2. It might be helpful to have a bit more discussion about the other data (e.g., the change-of-mind locked data) aside from the first frame of evidence, in addition to the figures that are presented. For example, is there anything worth discussing in Fig 1C and 1F?**

We have expanded our discussion of the data in Fig 1C in the results section as follows (Lines 140-147):

*‘This revealed clear patterns in the luminance residuals for change of mind trials. Evidence in early time windows favoured the initial response, whilst evidence in later time windows favoured the final response, replicating previous findings [3–5]. This can be most clearly observed in Fig 1C, where the psychophysical kernels for ‘corrected error’ and ‘spoilt correct’ trials slowly shift from favouring one choice outcome to the other over the course of ~1 second. Interestingly, the kernels both remain significantly different from zero even after the change of mind was enacted. This is likely due to the exclusion of double-change-of-mind trials from our analyses. That is, additional evidence in favour of the revised response may have been needed in order to avoid another secondary change-of-mind from occurring, and trials with secondary changes of mind were excluded from these plots.’*

We now also discuss Fig 1F in the Results section and highlight a potential point of confusion which could arise when inspecting those data (Lines 166-170):

*‘Plotting the residual stimulus fluctuations for fast and slow corrected errors and fast and slow spoilt responses revealed that changes of mind occurred more slowly after the initial response when the first frame of evidence strongly supported the initial decision (Fig. 1 D-F). Note, at around -500 ms in Fig 1F it may appear as if there is actually stronger evidence in favour of the initial response for fast change-of-mind trials, as compared to slow change of mind trials. However, this is simply due to a shifting of the relative timing of kernels, given the underlying differences in change-of-mind response time (see Fig 2F for a model-based recreation of this).’*

**R2C3. t-tests are conducted in Figure 1, but more detail could be provided as to how those are calculated. Are they based on participant mean scores for residual evidence at each point?**

These t-tests were conducted on the residual luminance values for each trial type, pooled across participants. One obvious limitation of this pooled analysis approach is that it fails to account for the effects of individual variation in the experimental effects. However, the aim of this analysis was simply to help illustrate the time points where there were clear differences in the kernels. For our main analysis we report more fine-grained mixed-effects model results, which explicitly model individual variation. We now clarify how the t-tests were conducted in the caption of Figure 1 (Lines 193-195).

*‘For illustrative purposes, t-tests (alpha level of .01) were also conducted to compare levels of residual evidence between specific trial types, at each frame. Residual evidence values were pooled across participants for these tests.’*

**R2C4. line 194-195 has a typo: "As such, change-of-mind decisions depend entirely on the quality of post-decisional.**

We appreciate the Reviewer drawing this typo to our attention and have fixed it as follows (Line 222-223):

*‘As such, change-of-mind decisions depend entirely on the quality of post-decisional evidence.’*

**R2C5. typo, line 383: "were excluded from all analyse."**

We appreciate the Reviewer drawing this typo to our attention and have fixed it as follows (Line 473-474):

*‘…were excluded from all analyses.’*
