# Supplementary material for: An initial ‘snapshot’ of sensory information biases the likelihood and speed of subsequent changes of mind
Source: PLoS Comput Biol. 2022 Jan 13;18(1):e1009738. doi: 10.1371/journal.pcbi.1009738 (PMC8757993; doi:10.1371/journal.pcbi.1009738)
Supplement: Attachment — Submitted filename: Turner_Response_To_Reviewers.docx [file pcbi.1009738.s008.docx]

We thank the Editor and Reviewers for their feedback on our revised manuscript entitled “**An initial ‘snapshot’ of sensory information biases the likelihood and speed of subsequent changes of mind**”. We have responded to all Reviewer comments and suggestions, and are confident that we present a much improved manuscript.

In this response letter, Reviewer comments are in bold, and our responses are in regular font. Quoted sections from the manuscript are in italics. For clarity and cross-referencing purposes, we have coded each of the Reviewers’ comments (e.g., R1C1, R2C2 etc.). Line numbers are used to indicate the location of specific changes in the revised manuscript. All changes in the revised manuscript have been highlighted in yellow.

**Reviewer #1**

**The authors have made a very good effort to address my initial sets of comments and most of the issues I raised are now resolved. I still believe there are some points regarding the primacy effect that need to be addressed however. I appreciate the authors are not claiming that only the first-frame of evidence influenced the occurrence of changes of mind (fortunately). However, as it seems it is the main point that they are focussing on and which justifies the novelty of the paper (as clearly emphasized in the title and abstract), I still find that some analysis are missing to understand the relevance of this effect for changes of mind. I agree with the authors it is novel and worth reporting, however I think it is equally important not to be misleading in the interpretation of the results and to report clearly the relative importance of this primacy effect compared in the evidence accumulation process leading to changes of mind. I detail below how I think the authors should address this.**

We thank the Reviewer for their feedback and are pleased that they are generally satisfied with our initial revisions. We now more clearly state the relative importance of the primacy effect, as compared to later evidence integration, in our revised manuscript. In this revision we have further expanded our discussion of this to better clarify this point.

Below, we address the Reviewer’s specific comments in turn and outline the modifications we have made.

**R1C1. I appreciate the authors have run additional analysis confirming that the mean pre-response evidence predicted the probability and latency of changes of minds. I think this is an important result that should be moved from the S4 appendix to the main text.**

We have moved the description of these analyses into the main text. Please note that we have left Fig S7, which shows the predicted marginal effects for the mean pre-response evidence models, in the Supplements. We have chosen to do this because the same figure showing the predicted marginal means for the primary analysis in our manuscript (Fig S1) is also reported in the Supplements rather than the main text. However, if the Reviewers and Editors feel these figures would be better placed in the main text, we are happy to make that change.

The relevant portion of the revised Results section now reads as follows (Lines 204-219):

*‘In addition to the main analyses reported above, we also examined the overall effect of pre-response evidence on the speed and likelihood of changes of mind. In particular, we fit a generalized linear mixed effects model to predict the probability of a change of mind occurring from the average pre-response evidence. Note, when calculating pre-response residual evidence, we excluded the initial frame. This allowed us to investigate the impact that subsequent sensory evidence had on change-of-mind behaviour, to get a general sense of the relative importance of the ‘primacy effect’ we report above. This analysis revealed a significant interaction between initial decision accuracy and pre-response evidence strength (likelihood ratio test, χ²(1) = 192.36, p < 2.20 x 10^-16^; see Fig. S7A). This indicates that when pre-response evidence (excluding the initial frame) strongly supported participants’ initial decisions, then changes of mind were less likely to occur. To formally test the effect of pre-response evidence on change of mind latency, we fit a linear mixed effects model to predict the speed with which changes of mind occurred. This revealed a significant interaction between initial decision accuracy and subsequent pre-response evidence strength (likelihood ratio test, χ²(1) = 82.36, p = 2.20 x 10^-16^; see Fig. S7B).*

*Importantly, these analyses show that the initial frame of evidence is neither the sole, nor necessarily the best, predictor of change of mind behaviour. …’*

Note, in the remainder of this second paragraph, we formally compare the relative importance of early (0-200ms) and late (200-400ms) evidence, on change of mind behaviour (as suggested by Reviewer 1). This paragraph is reported in full in our response to the following comment, and helps to further clarify the impact of sensory evidence across time points on change-of-mind behaviour.

Finally, when reporting our main analysis, we signpost these additional analyses as follows (Lines 157-159):

*‘However associations between stimulus evidence strength and decision behaviour were by no means restricted to just the first frame (see below for additional analyses regarding the effects of subsequent evidence).’*

**R1C2. I think an additional analysis should be run to compare the predictive power of the initial frame of evidence and the rest of the pre-response window. According to figure S7, it looks like the mean of the pre-response window is a better predictor of occurrence of changes of minds than the initial snapshot singled out by the authors. However, this should be tested and quantified. As the mean of the whole pre-response window is not comparable to evidence presented in only one frame, I would suggest using a different approach than computing the mean pre-response evidence. One possibility would be to run the same GLM analysis comparing the predictive power of the initial snapshot, and then another sample 100 or 200ms later, correcting for multiple comparison. Another possibility would be to compute the mean over the 0-200ms time window and compare it to the 200-400ms for instance.**

We agree that it is interesting to consider how the predictive power of information varies across time. Below we run and report the suggested analysis comparing the predictive power of mean evidence between 0-200ms and 200-400 ms.

However, before reporting this, we think it is important to distinguish between two concepts: the ‘predictive power’ of evidence at specific time points, and the ‘weight’ given to information at specific time points. First, let us consider single decisions that are not followed by a change of mind. Given the dynamics of bounded evidence accumulation, even when information is weighted constantly (as in the standard diffusion model), across repeated decisions the predictive power of sensory evidence will gradually decrease over time. This is because, as time passes, more and more decisions will be finalised (i.e. decision processes will reach the decision threshold). Once a decision is finalised, each subsequent frame of evidence within that trial will be unable to influence the decision outcome, resulting in a gradual decrease in predictive power across trials. Critically however, the gradual decrease in predictive power does not reflect a gradual decrease in evidence weighting, but rather the averaged effect of step-wise drops in evidence weighting that occur with each finalised decision.

Importantly, when changes of mind are allowed to occur, ‘post-decisional’ frames of evidence will be able to influence the likelihood and speed of changes of mind, even if they can no longer influence the initial decision. In this situation, the degree to which each frame of evidence can predict change of mind behaviour will vary depending on both the number of initial decisions and the number of changes of mind that have been finalised. Given this complexity, we believe it is important to be mindful that changes in predictive power cannot necessarily be interpreted directly as changes in evidence weighting.

Despite this complexity, we agree that it is interesting and worthwhile to examine differences in the predictive power of evidence across different timepoints. We have therefore run the suggested analysis comparing the predictive power of mean evidence between 0-200 ms and 200-400 ms. Specifically, we fit two generalized linear mixed effects models to predict the probability of a change of mind occurring from the average evidence in these two time windows. The structure of these models was otherwise identical to those used in the primary analysis of the paper. To compare these two *non-nested* generalized linear mixed-effects models, we compared their AIC and Marginal/Conditional R^2^ values (because the models are non-nested, a likelihood ratio test could not be used as in the main analyses). Comparing the models, we found that mean evidence between 200-400ms was a better predictor of change of mind likelihood than mean evidence between 0-200ms (AIC of 14961 vs 15039, Marginal R^2^/Conditional R^2^ 0.370/0.372 vs. 0.364/0.367). This demonstrates that evidence in this later time window was better predictive of change-of-mind behaviour.

Importantly, this result should only be considered under the current experimental conditions. This is because changes in response-speed pressure, for example, would shift the average time at which decision processes are transitioning from the ‘pre-decisional’ to ‘post-decisional’ period, and thus influence how predictive power for change of mind behaviour varies across time.

In the revised manuscript, we have incorporated this new result into our discussion of the relative impact of stimulus evidence over time. More generally, we have revised this section to better clarify the relative impact of initial evidence on change-of-mind behaviour. The revised section now reads as follows (Lines 218-230):

*‘Importantly, these analyses show that the initial frame of evidence is neither the sole, nor necessarily the best, predictor of change of mind behaviour. Indeed, under the current experimental conditions, mean evidence between 200-400 ms after stimulus onset was a better predictor of change of mind likelihood than mean evidence between 0-200ms (non-nested GLMM comparison: AIC 14961 vs. AIC 15039; Marginal R^2^/Conditional R^2^ 0.370/0.372 vs. 0.364/0.367, see Supplement 6 for model parameter estimates). Considering the patterns revealed by the psychophysical reverse correlation analysis, as well as the results of these additional analyses, it is clear that changes-of-mind arise due to stereotyped changes in the sensory evidence across many timepoints. Clearly, most frames of evidence are able to contribute to these decisions in some way, with late arriving evidence potentially have a greater impact than early evidence. However, as we demonstrate below, the fact that the very earliest frame of evidence can even influence change-of-mind behaviour at all, regardless of the magnitude of this effect, is theoretically interesting and reveals important details about the structure of the processes underlying change of mind decisions.’*

Supplement 6 is included on the following page for reference.

***S6 Appendix. Parameter estimates for 1-200 ms and 200-400 ms mean evidence models.***

***Table S3.*** *Mixed-effects models estimates*

|  | *Change-of-mind likelihood*  *(Mean evidence 1-200ms)* | | | | *Change-of-mind likelihood*  *(Mean evidence 200-400ms)* | | | |
| --- | --- | --- | --- | --- | --- | --- | --- | --- |
| *Predictors* | *Odds Ratios* | | *CI* | *p* | *Odds Ratios* | *CI* | *p* | |
| *(Intercept)* | *1.78* | | *1.56 – 2.02* | ***<.001*** | *1.68* | *1.49 – 1.90* | ***<.001*** | |
| *Accuracy(1)* | *0.05* | | *0.05 – 0.06* | ***<.001*** | *0.05* | *0.5 – 0.06* | ***<.001*** | |
| *Evidence* | *6.26* | | *3.66 – 10.7* | ***<.001*** | *13.40* | *7.90 – 22.71* | ***<.001*** | |
| *Accuracy*Evidence* | *0.02* | | *0.01 – 0.05* | ***<.001*** | *0.01* | *0.00 – 0.01* | ***<.001*** | |
| *Marginal R^2^/Conditional R^2^* | | *0.364 / 0.367* | | |  | *0.370 / 0.372* | |  |

**R1C3. I thank the authors for their detailed reply and additional results presented. One thing that I think might be worth mentioning in the discussion about the generalisability of the effect is the issue of temporal expectancy. As in the design, the stimulus appeared always at predictable time following the start of the trial, could it be that participants learn to focus their attention to when the initial frame of the stimulus would appear?**

We thank the Reviewer for this suggestion and agree that it is useful to mention this possibility. They are correct that stimulus onset times were predictable, and it is possible that this may have led participants to temporally expect and attend to early information. However, while stimulus predictability may affect initial decisions, it is not clear why this would necessarily also enhance an effect on change-of-mind decisions, as we observe here. Moreover, even if stimulus predictability did draw attention to the onset of the stimulus, this does not challenge our overarching argument, which is that the very existence of this primacy effect on change-of-mind is theoretically interesting, given the prevailing dominant model (Resulaj et al., 2009). We agree, however, that it is useful to mention this possibility, to prompt future research.

We now mention the issue of temporal expectancy in the Discussion as follows (Lines 348-362):

*‘Importantly, it may be the case that the ‘primacy effect’ we observed is only detectable in the context of low-level visual tasks which require minimal temporal integration. Similarly, the fact that the timing of stimulus onset was predictable in the present study, may have led participants to attend more to early information. If future work identified a differential use of information for change of mind decisions, depending on the features of evidence integration, and/or the predictability of stimulus timing, this would in itself advance our understanding of decision dynamics.’*

**R1C4. Thanks for analysing the proportion of trials in which the signal favoured the incorrect response. I agree the proportion is small enough to have a negligible effect but it is worth reporting it still in the supplementary material.**

We now report this in Supplement 5 of the revised manuscript as follows:

***‘S5 Appendix. Auxiliary analysis of trials in which the signal favoured the incorrect response***

*We conducted an auxiliary analysis of the proportion of trials in which, by chance, the overall mean signal was in favour of the incorrect response, to rule out that these trials were driving our results in some way. When averaging across all timepoints, the proportion of trials in which this occurred was 0.00067 (i.e. 13 trials). However, one reason this proportion is so small is because it is calculated across the whole trial, which includes the full 1 second post-decisional time period. Given this, we ran an additional analysis on the first 600ms of each trial. We chose this time window since it approximates the maximum duration that participants would have been sampling evidence for their initial decision, given the estimated kernels in Figure 1B. Even in this time window the proportion of trials was only 2.98%. Given that the overall change of mind rate was 23.91% (18.53% corrected errors), it is unlikely that trials in which mean evidence was favouring the incorrect response were driving this behaviour. The psychophysical reverse correlation analyses further serve to demonstrate that this is unlikely. These analyses show that changes of mind were driven by stereotyped changes in information across time, where initial information supports once choice and later information supported the other. Finally, we note that random fluctuations in favour of the incorrect response are a feature of computational models we employed. This serves as further evidence that the observed effects are unlikely to be driven by trials in which mean evidence favoured the incorrect response.’*

**R1C5. Thanks for reporting this interesting finding of the model with the down weighting of the initial evidence. I think that strengthen the point made in the paper and is well highlighted in the updated part of the discussion.**

We thank the Reviewer for prompting this helpful additional analysis.

**Reviewer #2**

**The authors have done an excellent job addressing the comments made on the initial submission, and I think this paper is a strong contribution to the field.**

We thank the Reviewer for their positive feedback and are pleased that they are satisfied with our initial revision.

**I only have a couple minor comments:**

**R2C1. The figures included in this version are quite blurry - hopefully this is something the authors can fix in the final submission.**

We thank the Reviewer for noting this. We have increased the resolution of the figures and made sure they comply with the journals figure guidelines, by using the PACE image tool.

**R2C2. line 285 typo: "relative rarity of these trial" should be "trials"**

We thank the Reviewer for bringing this to our attention and have fixed the typo.

**R2C3. for the added paragraph about continuous responses vs. discrete responses in the discussion, it might be worth also noting that continuous responses involve more physical effort which may be a contributing factor to the likelihood and nature of changes of mind (several studies showing this are already cited in this paragraph).**

We agree that this is an important detail to highlight. We have expanded the relevant section as follows (Lines 420-431):

*‘We also chose to record responses in a binary rather than continuous fashion. This allowed us to precisely measure the onset times of change-of-mind responses, adding further constraint on the computational models. However, continuous response measures, such as movement trajectories, have been employed in a number of past studies [3,5,7,8] and have the advantage of allowing for changes of mind to be observed in a potentially more graded fashion. Moreover, physical effort costs are likely to differ for discrete and continuous responses, influencing the computations underlying change-of-mind decisions [7,8]. One question, which arises in light of these considerations, is whether the use of discrete response measures may have led to slightly different decision-making behaviour. For example, when continuous response measures are employed, participants may adopt different strategies because movements can be initiated before a strict decision has been finalized, and because they may be weighing up effort costs which evolve over time [7,8,22].’*

**R2C4. Regarding the discussion on temporal integration and the RDK tasks, it may be worth discussing or speculating on real-world examples where changes-of-mind may occur that do (or do not) involve temporal integration. In other words, regarding R1's concerns about generalizability, perhaps the initial snapshot of evidence is only useful in a subset of cases. Is it likely that many real-world behaviours involving changes of mind are covered by these cases?**

We agree that it may be useful to briefly illustrate examples of situations in which the degree of temporal integration, and hence the degree to which initial evidence can influence change-of-mind behaviour, will likely vary. To this end, we have added the following sentences to the Discussion on temporal integration (Lines 351-358):

*‘If future work identified a differential use of information for change of mind decisions, depending on the features of evidence integration, and/or the predictability of stimulus timing, this would in itself advance our understanding of decision dynamics. This would also help to clarify the degree to which change-of-mind behaviour is influenced by initial sensory evidence under different environmental conditions or decision scenarios – something which is of relevance when considering real-world decisions which rely on either rapid or more sustained integration of sensory information (e.g., gauging the color of a traffic light in clear conditions compared to judging the speed of approaching traffic in heavy rain).’*
